# Application of Digital Holographic Imaging to Monitor Real-Time Cardiomyocyte Hypertrophy Dynamics in Response to Norepinephrine Stimulation

**DOI:** 10.3390/app14093819

**Published:** 2024-04-30

**Authors:** Wahida Akter, Herman Huang, Jacquelyn Simmons, Alexander Y. Payumo

**Affiliations:** Department of Biological Sciences, San Jose State University, San Jose, CA 95192, USA

**Keywords:** neonatal rat, heart, cardiomyocyte, hypertrophy, dynamics, norepinephrine, cell culture, digital holographic imaging, live cell imaging

## Abstract

Cardiomyocyte hypertrophy, characterized by an increase in cell size, is associated with various cardiovascular diseases driven by factors including hypertension, myocardial infarction, and valve dysfunction. In vitro primary cardiomyocyte culture models have yielded numerous insights into the intrinsic and extrinsic mechanisms driving hypertrophic growth. However, due to limitations in current approaches, the dynamics of cardiomyocyte hypertrophic responses remain poorly characterized. In this study, we evaluate the application of the Holomonitor M4 digital holographic imaging microscope to track dynamic changes in cardiomyocyte surface area and volume in response to norepinephrine treatment, a model hypertrophic stimulus. The Holomonitor M4 permits non-invasive, label-free imaging of three-dimensional changes in cell morphology with minimal phototoxicity, thus enabling long-term imaging studies. Untreated and norepinephrine-stimulated primary neonatal rat cardiomyocytes were live-imaged on the Holomonitor M4, which was followed by image segmentation and single-cell tracking using the HOLOMONITOR App Suite software version 4.0.1.546. The 24 h treatment of cultured cardiomyocytes with norepinephrine increased cardiomyocyte spreading and optical volume as expected, validating the reliability of the approach. Single-cell tracking of both cardiomyocyte surface area and three-dimensional optical volume revealed dynamic increases in these parameters throughout the 24 h imaging period, demonstrating the potential of this technology to explore cardiomyocyte hypertrophic responses with greater temporal resolution; however, technological limitations were also observed and should be considered in the experimental design and interpretation of results. Overall, leveraging the unique advantages of the Holomonitor M4 digital holographic imaging system has the potential to empower future work towards understanding the molecular and cellular mechanisms underlying cardiomyocyte hypertrophy with enhanced temporal clarity.

## 1. Introduction

Cardiomyocyte hypertrophy—an increase in the size of individual heart muscle cells—is a hallmark feature of many cardiovascular diseases that may arise from a number of pathological conditions including hypertension, myocardial infarction, and valve dysfunction. While initially considered an adaptive response to hemodynamic stress or injury, sustained myocardial hypertrophic growth often progresses to heart failure, a leading cause of morbidity and mortality worldwide [1]. Pathological hypertrophy is often accompanied by adverse structural and functional alterations in heart function due to fibrosis, impaired contractility, and arrhythmogenesis [2]. Pathological hypertrophy often precedes the development of heart failure and other adverse cardiovascular events [3]. Understanding the dynamics of pathological cardiac hypertrophy at the cellular level may lead to the development of novel treatment strategies to mitigate disease progression and improve patient outcomes.

While in vivo studies have provided invaluable insights into the physiological signals underlying pathological hypertrophy of the heart, in vitro primary cardiomyocyte culture models have contributed significantly to our understanding of the cellular and molecular mechanisms that drive cardiomyocyte hypertrophic responses. For instance, studies have employed primary cardiomyocyte cultures to explore the effects of various hypertrophic stimuli, such as mechanical stretch, neurohormonal factors, and growth factors [4,5]. Additional studies have revealed key intracellular signaling cascades, including the calcineurin-NFAT and MAPK pathways, that contribute to hypertrophic growth responses [6,7]. Overall, in vitro primary cardiomyocyte culture models have served as valuable tools for dissecting the multifaceted processes that drive cardiomyocyte hypertrophic growth and greatly complement in vivo studies. However, despite the advantages of in vitro primary cardiomyocyte culture models, the current strategies used to assess cardiomyocyte size and hypertrophic growth are limited to static end-point measurements [8-10]. Surface area assessments of cardiomyocytes in two dimensions [11] overlook alterations in cell thickness and primarily focus on cell spreading. Coulter counters [8] and flow cytometry [9] offer measurements at the population level but lack the ability to capture individual cell dynamics. Additionally, three-dimensional confocal imaging [10] often requires cell fixation and optical sectioning using high-intensity lasers, thereby limiting the prolonged observation of live cardiomyocytes. Therefore, the temporal dynamics of cardiomyocyte hypertrophic responses have remained highly understudied.

The Holomonitor M4, a commercially available digital holographic imaging microscope small enough to fit within standard cell culture incubators, permits the non-invasive, label-free, long-term quantitative three-dimensional imaging of individual living cells [12]. An interference pattern created between a sample and a reference beam generates phase shift data, which are then reconstructed to visualize the cultured cells in three dimensions. The goal of this study is to evaluate if the Holomonitor M4 imaging system can be applied to quantitatively track the three-dimensional dynamics of cardiomyocyte hypertrophic responses in real-time. The powerful HOLOMONITOR App Suite analysis software facilitates quantitatively monitoring single-cell behaviors and morphology simultaneously. We aim to determine if these unique imaging and tracking capabilities can be leveraged to provide further insights into the temporal aspects of cardiomyocyte hypertrophic growth responses.

## 2. Materials and Methods

### 2.1. Animal Breeding

Neonatal rat pups were generated through natural matings approved by the Institutional Animal Care and Use Committee (IACUC) protocol 1072 at San Jose State University.

### 2.2. Cardiomyocyte Isolation and Culture

Rat pups from postnatal day 1 (P1) to P3 Sprague Dawley breeding pairs were euthanized by decapitation while under manual restraint. Following euthanasia, their hearts were collected, and the aorta of each heart was clamped shut using forceps. Subsequently, the coronary vessels were reverse-perfused with a solution of cardiomyocyte isolation buffer (CIB; 120 mM sodium chloride, 15 mM potassium chloride, 0.6 mM monopotassium phosphate, 0.6 mM sodium phosphate dibasic heptahydrate, 1.2 mM magnesium sulfate heptahydrate, 10 mM HEPES, 4.6 mM sodium bicarbonate, 30 mM taurine, 10 mM 2,3-butanedione monoxime, 5.5 mM glucose) supplemented with 0.4 mM EGTA by inserting a 20 G needle into the left ventricle to clear out blood and improve digestion efficiency. Following perfusion, the ventricles were separated, diced, and placed into a digestion solution comprising 2 mg/mL collagenase II (LS004176, Worthington Biochemical, Lakewood, NJ, USA) and 0.3 mM calcium chloride in CIB. The tissues were digested at 37 °C with gentle agitation for about 2 h, with dissociated cells being collected every 15 to 30 min until complete digestion was achieved. The collections were combined and kept on ice to minimize the activity of collagenase. Following digestion, the dissociated cells were centrifuged gently for 5 min at 200× *g*. They were then successively suspended in wash buffer 1 (CIB, 10% FBS, 0.64 mM calcium chloride), wash buffer 2 (CIB, 10% FBS, 1 mM calcium chloride), and finally in plating media containing 10% FBS in DMEM/F12 (SH30023.01, Cytiva, Marlborough, MA, USA) supplemented with 100 μg/mL Primocin (ant-pm, InvivoGen, San Diego, CA, USA). The cells were subsequently filtered through a 70 μm cell strainer (258368, Nest Scientific, Woodbridge, NJ, USA) into a 6-well plate for pre-plating. This pre-plating step was conducted at 37 °C in a 5% CO_2_ incubator for 1 to 2 h to deplete non-cardiomyocyte populations through selective attachment. Following the pre-plating step, the culture medium along with the remaining cells were gathered, quantified, and subsequently distributed into 6-well plates (with 800,000 cells per well) that had been previously coated with a solution of 1% weight/volume gelatin (G9391, Sigma-Aldrich, St. Louis, MO, USA) for at least 30 min. Cardiac cells were left in plating media overnight to facilitate the attachment of cardiomyocytes to the 6-well plates.

### 2.3. Chemical Treatments

After the 16 to 24 h overnight adherence period, cardiac cultures were rinsed three times with PBS to eliminate serum remnants and then transitioned to a serum-free culture medium comprising MCDB107 (E3000, United States Biological, Salem, MA, USA) supplemented with 1X insulin, transferrin, and selenium (ITS, 400-145, GeminiBio, West Sacramento, CA, USA) along with 100 μg/mL Primocin. Cultures were either left untreated or stimulated with 10 μM norepinephrine (A0937, Sigma) for 24 h to induce cardiomyocyte hypertrophy. A 1000X 10 mM stock of norepinephrine was prepared fresh prior to each experiment and was dissolved directly in the serum-free culture medium.

### 2.4. Digital Holographic Time-Lapse Imaging

A Holomonitor M4 digital holographic microscope was purchased from Phase Holographic Imaging (PHI, Lund, Sweden) and placed directly within a Heracell 150 CO_2_ incubator (Thermo Scientific, Waltham, MA, USA). For digital holographic imaging experiments, rat cardiac cells were cultured and treated with chemicals in 6-well plates (83.3920, Sarstedt, Nümbrecht, Germany) and then covered with HoloLids (PHI, Sweden) to stabilize imaging conditions. The cultures were then transferred into a 37 °C and 5% CO_2_ incubator to acclimatize for an additional 1 h and reduce condensation on the well bottoms. Then, 10 imaging positions were selected per treatment using the HOLOMONITOR App Suite software. This process took approximately 30 min. After the position selection, live rat cardiac cultures were imaged on the Holomonitor M4 for 24 h, with time points taken every 20 min at 37 °C and 5% CO_2_.

### 2.5. Single-Cell Tracking of Surface Area and Optical Volume Dynamics

Time-lapse recordings were analyzed in the Holomonitor App Suite software version 4.0.1.546 (PHI, Sweden) using the “In-depth Analysis: Single Cell Tracking” module to segment, trace, and quantify single-cell dynamics. The following segmentation thresholds were applied: auto-minimum error method, adjustment 134, and minimum object size 17. Cardiomyocytes were qualitatively distinguished from non-cardiomyocytes based on their larger size and limited motility as previously described [13]. Care was taken to track well-isolated cells that remained unambiguous throughout the entire imaging duration. Attention was paid to ensuring that all the analyzed cells were adequately well isolated, and the precision of cell segmentation was manually confirmed for each imaging frame. Cells that clustered or where segmentation was ambiguous at any point during the experiment were omitted from the analysis. After cell identification, surface area and optical volume measurements were recorded at the desired time intervals.

### 2.6. Statistical Analysis

**Figure 1.**
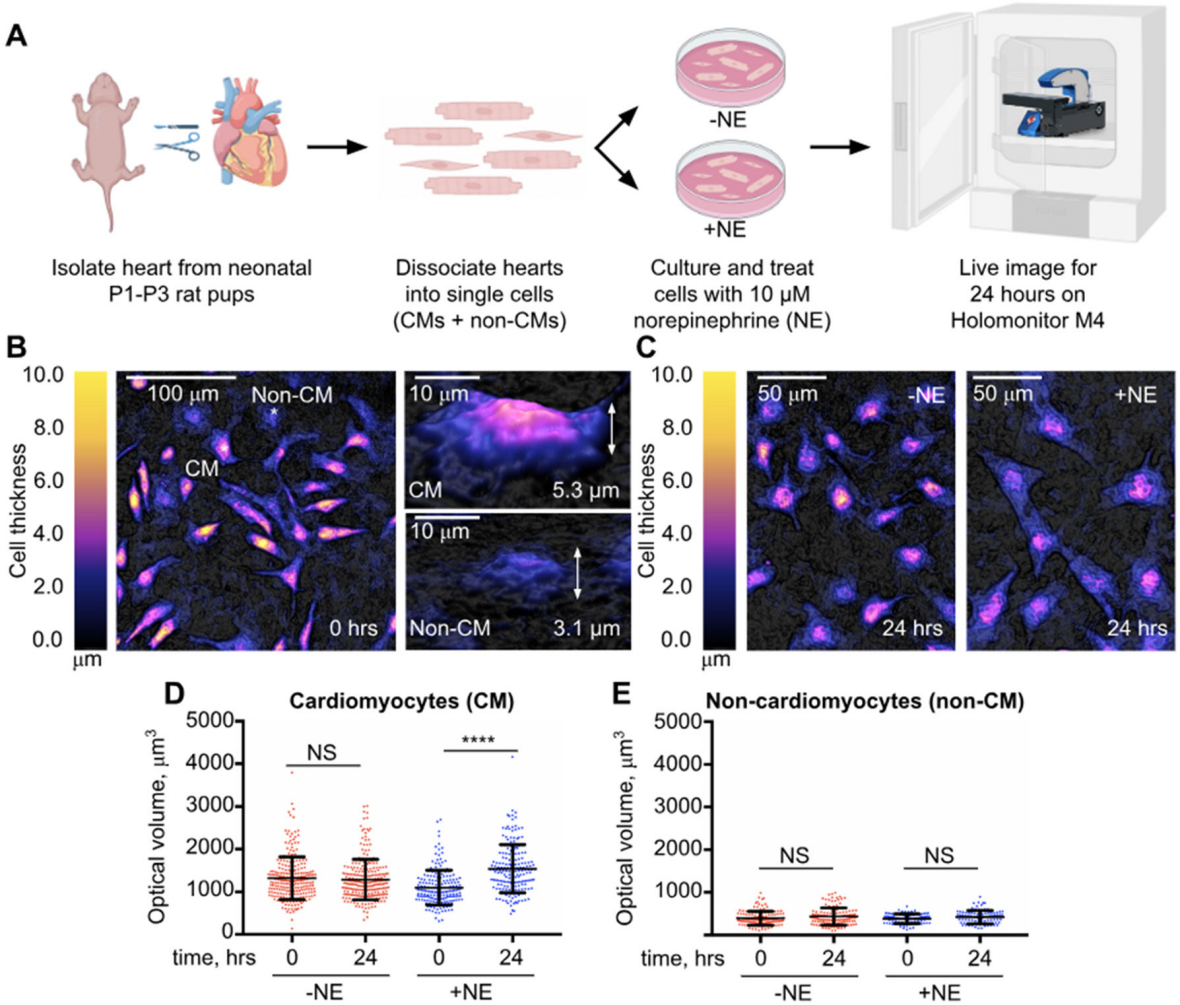
Quantification of cardiomyocyte optical volumes in response to 24 h stimulation with 10 μM norepinephrine. (**A**) Overview of experimental design. Cardiac cultures derived from the hearts of postnatal day 1 (P1) to P3 rat pups cultured with and without 10 μM norepinephrine (NE) treatment and then visualized for 24 h on the Holomonitor M4 digital holographic imaging microscope. (**B**) Representative three-dimensional reconstructions of cultured cardiomyocytes (CM) and non-cardiomyocytes (non-CM) as visualized on the Holomonitor M4 at the beginning of our time-lapse imaging experiments at 0 h (0 hours). CMs were identified based on their larger size and overall thickness. (**C**) Representative three-dimensional reconstructions of cultured CMs untreated or stimulated with 10 μM NE at the end of our time-lapse imaging experiments (24 h). NE-treated CMs exhibited dramatic changes in morphology including spreading and the development of protrusions. (**D**) Measured optical volumes of untreated (*n* = 256) and NE-stimulated (*n* = 187) CMs at 0 h and 24 h of live-imaging on the Holomonitor M4 quantified from 4 independent cardiac cultures derived from P1–P3 rat hearts. (**E**) Measured optical volumes of untreated (*n* = 140) and NE-stimulated (*n* = 90) non-CMs at 0 and 24 h of live-imaging on the Holomonitor M4 quantified from 3 independent cardiac cultures derived from P1-P3 rat hearts. Values are reported as mean ± standard deviation. One-way ANOVA, Tukey’s multiple comparisons test; ****, *p* < 0.0001; NS, not significant.

**Figure 2.**
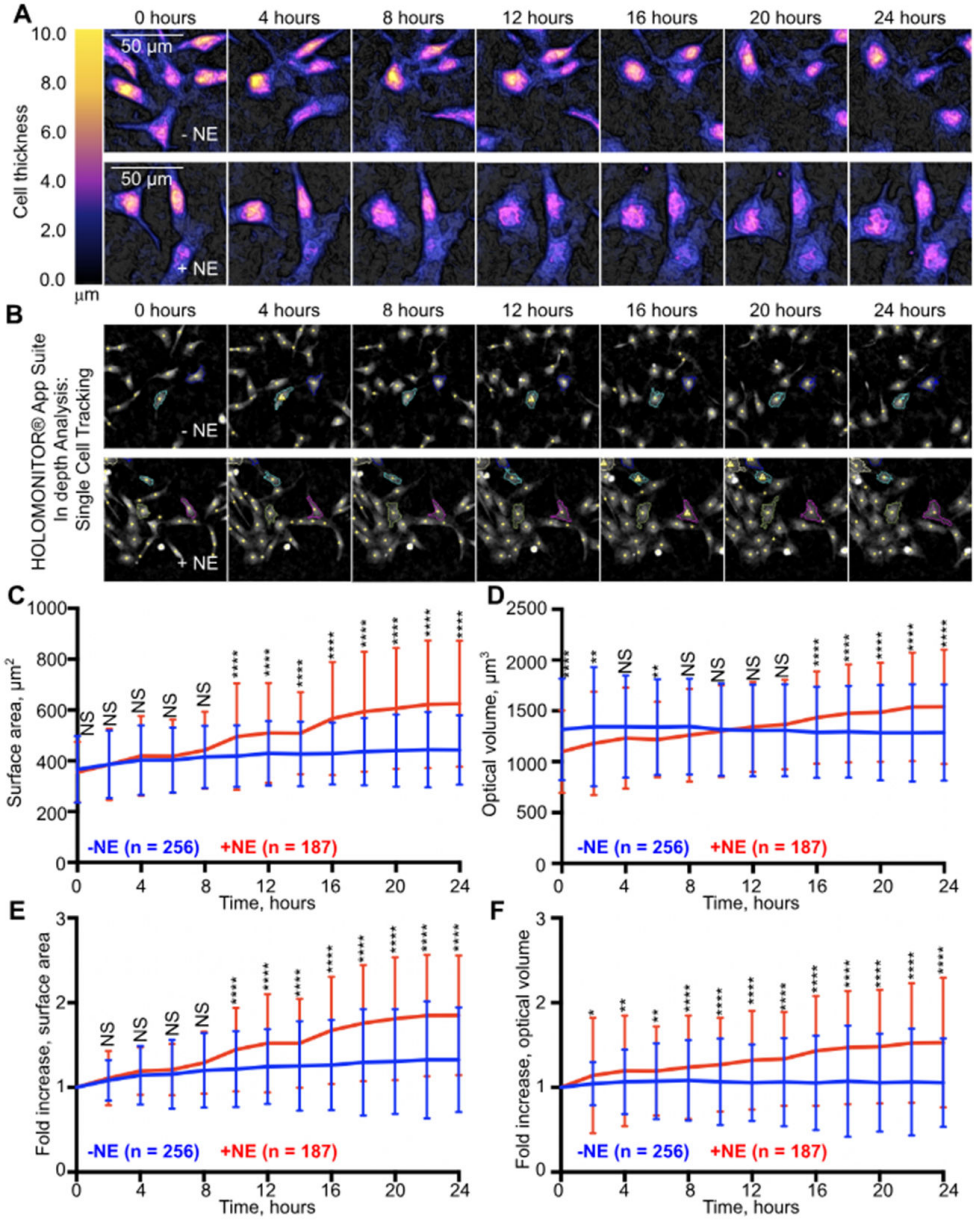
Single–cell tracking of cardiomyocyte surface area and optical volume dynamics during 24 h treatment with 10 mM norepinephrine. (**A**) Representative panels of three-dimensional reconstructions of cultured cardiomyocytes (CM) untreated or stimulated with 10 μM norepinephrine (NE) over 24 h of live imaging. (**B**) Representative panels depicting image segmentation and identification of well-isolated CMs using the HOLOMONITOR App Suite software and the Single Cell Tracking module. Drastic changes in cell volumes are flagged by the software (yellow triangles containing exclamation points) to assist with the validity of cell identification tracking. Raw surface area (**C**) and optical volume (**D**) dynamics of untreated (*n* = 256) and NE-stimulated (*n* = 187) cells over the course of 24 h imaging. Normalized surface area (**E**) and optical volume (**F**) dynamics were determined by dividing the value obtained at each time point by the initial value measured in cells at the start of the imaging experiment (0 h). Cellular measurements are pooled from the analysis of cultures from 4 independent cardiac culture preparations from P1–P3 rat hearts. Multiple *t*-test analysis was performed to determine statistical difference between untreated and NE-stimulated cells at each time point using the two-stage step-up method of Benjamini, Krieger, and Yekutieli with FDR set at 1%: *, *p* < 0.05; **, *p* < 0.01; ****, *p* < 0.0001; NS, not significant.

Statistical analyses were conducted by utilizing GraphPad Prism software version 7.0e (GraphPad Software Inc., Boston, CA, USA). Detailed information regarding the statistical methods employed and the sample sizes assessed can be found in the respective figure legends. In short, the data represented in Figure 1D,E were analyzed using one-way ANOVA followed by Tukey’s multiple comparison tests. The data represented in Figure 2C-F were analyzed using multiple *t*-test analyses to determine the statistical differences between untreated and NE-stimulated cells at each time point using the two-stage step-up method of Benjamini, Krieger, and Yekutieli, with FDR set at 1%.

## 3. Results

### 3.1. Validation of the Holomonitor M4 Digital Holographic Imaging System to Detect Norepinephrine-Induced Cardiomyocyte Hypertrophic Growth

Chronic activation of the sympathetic nervous system can contribute to left ventricular cardiac hypertrophy [14]. Consistent with this, direct stimulation of isolated primary rat cardiomyocytes with the sympathetic neurotransmitter norepinephrine induces hypertrophy in serum-free culture [15]. We, therefore, sought to use this well-established in vitro model of cardiomyocyte hypertrophy to determine the effectiveness of the Holomonitor M4 digital holographic imaging microscope in detecting norepinephrine-dependent increases in cell volume that should accompany bona fide hypertrophic growth (Figure 1).

We isolated hearts from postnatal day 1 (P1) to P3 rat pups, dissociated the tissue into single cells, cultured the cells with and without the presence of 10 μM norepinephrine, and then live imaged the cells directly on the Holomonitor M4 for 24 h (Figure 1A). Cardiac cultures from neonatal rat hearts are often contaminated with other cell types in addition to cardiomyocytes, despite methods used to increase purity [16,17]. Our lab has recently observed that α-actinin-positive cardiomyocytes can be distinguished from non-cardiomyocyte cells imaged on the Holomonitor M4 based on their overall larger size and slower motility [13] (Figure 1B). Using these criteria, we were able to readily identify cardiomyocytes in our cardiac cultures, both with and without norepinephrine treatment (Movies S1 and S2). As expected, the norepinephrine-treated cardiomyocytes exhibited significant spreading and formed cellular protrusions by the end of the 24 h treatment relative to the untreated controls (Figure 1C), which is consistent with previous observations [15].

A key technical advantage of the Holomonitor M4 digital holographic imaging system is its ability to reconstruct the three-dimensional morphologies of cultured cells. A two-dimensional increase in surface area due to cell spreading is not indicative of a three-dimensional increase in cell volume. Therefore, we utilized the HOLOMONITOR App Suite software and its Single Cell Tracking module to quantify the three-dimensional optical volumes of individual cardiomyocytes and non-cardiomyocytes at the start (0 h) and finish (24 h) of our live imaging experiments (Figure 1D,E). At 0 h, the average optical volumes of the individual untreated cardiomyocytes (*n* = 256) were 1380 ± 501 μm^3^ and that of the norepinephrine-treated cardiomyocytes (*n* = 187) were slightly decreased at 1074 ± 501 μm^3^ (Figure 1D). However, at 24 h, the average optical volumes of the individual untreated cardiomyocytes (*n* = 256) were 1341 ± 473 μm^3^ and that of the norepinephrine-treated cardiomyocytes (*n* = 187) were significantly increased to 1492 ± 501 μm^3^. These results show that during the 24 h imaging duration, the untreated cardiomyocytes did not exhibit any meaningful increase in optical volume (0.98-fold), while the volume of the norepinephrine-treated cardiomyocytes increased by approximately 1.39-fold. Non-cardiomyocytes, identified by their high motility and smaller size, did not exhibit any significant norepinephrine-dependent changes in optical volume at either the 0- or 24 h time point (Figure 1E), suggesting that stimulation with the neurohormone induced a cardiomyocyte-specific increase in cell volume.

### 3.2. Application of the Holomonitor M4 Digital Holographic Imaging System to Monitor Real-Time Cardiomyocyte Hypertrophic Growth Dynamics

Various technologies are capable of quantitatively evaluating cardiomyocyte size, including Coulter counter analysis [8], flow cytometry [9], and confocal imaging [10]. However, they are limited in their ability to directly and non-invasively monitor the real-time dynamics of cardiomyocyte hypertrophic responses over extended durations. A major advantage of the Holomonitor M4 is that it utilizes a low-power 635 nm laser, which minimizes phototoxicity, making the instrument ideal for long-duration live-cell imaging [12]. Cardiomyocyte size is typically evaluated by an increase in two-dimensional surface area or three-dimensional volume. Therefore, we questioned if the Holomonitor M4 could be uniquely applied to quantitatively track dynamic changes in cardiomyocyte surface area and optical volume throughout our 24 h imaging experiments (Figure 2A).

The HOLOMONITOR App Suite analysis software allows for in-depth quantitative analysis of cell morphology and behavior. We used the Single Cell Tracking module to apply image thresholds to segment each frame of our time-lapse videos and identify individual cardiomyocytes (Figure 2B). We then manually selected well-isolated cardiomyocytes to follow and track throughout the entire 24 h imaging duration. We verified the accuracy of segmentation for each tracked cell in each imaging frame. This was assisted by the HOLOMONITOR App Suite software, which automatically flagged cells that exhibited dramatic changes in cell volume for closer inspection (Figure 2B). Cells that converged with other cells or those for which tracking became ambiguous at any point during the imaging period were omitted from analysis.

We first analyzed the surface area dynamics of both the untreated and norepinephrine-stimulated cardiomyocytes over the 24 h imaging period (Figure 2C). T-test comparisons were applied at each time point to determine instances when surface areas statistically differed between the two experimental conditions. This analysis suggested that norepinephrine increases cardiomyocyte surface area as early as 10 h after treatment. However, it is important to note that the untreated cardiomyocytes also exhibited a mild norepinephrine-independent 1.2-fold increase in surface area by the end of the imaging experiment. This could be due to the presence of cardiac fibroblasts in culture, which have been reported to increase cardiomyocyte surface area in vitro [18]. Therefore, it may be difficult to uncouple norepinephrine-dependent and independent effects on cardiomyocyte hypertrophy when measuring only surface area.

When we analyzed cardiomyocyte three-dimensional optical volume dynamics, we noticed that the untreated cells cultured in our serum-free conditions exhibited little change in their average optical volume over the entire imaging duration (0.98-fold), suggesting that optical volume may behave more stably in our experimental timeframe. However, the average starting optical volumes of the norepinephrine-treated cardiomyocytes were significantly decreased compared to that in the untreated control cells (Figures 1D and 2D). Therefore, we could not reliably evaluate when norepinephrine stimulation induced a statistically significant change in cardiomyocyte volume due to these differences in baseline measurements.

The Holomonitor M4 and HOLOMONITOR App Suite software permit longitudinal tracking of cardiomyocyte dynamics with single-cell resolution. This enabled us to normalize the size of each individual cardiomyocyte based on its initial value measured at the start of our imaging experiments (Figure 2E,F). This analysis revealed a clearer dynamic of norepinephrine-stimulated cardiomyocyte hypertrophy. T-test comparisons applied at each time point revealed statistically significant norepinephrine-dependent increases in optical volume as early as 2 h after the start of our imaging experiments (Figure 2F). Taken together, these results provide proof-of-principle evidence that the Holomonitor M4 digital holographic imaging system can be successfully applied to monitor dynamic changes in cardiomyocyte surface area and volume in response to a hypertrophic stimulus.

## 4. Discussion

In this study, we evaluated the application of a commercially available Holomonitor M4 digital holographic imaging microscope to detect dynamic changes in primary neonatal rat cardiomyocyte surface areas and optical volumes in response to norepinephrine stimulation leading to hypertrophic growth. The Holomonitor M4 utilizes a low-powered 635 nm laser to generate holograms for three-dimensional image reconstruction [19], promoting live-cell imaging over extended durations [20]. However, if phototoxicity is observed, it may be possible to acquire time-lapse images at longer time intervals to further minimize the risk.

This label-free imaging approach has great potential to characterize the cellular dynamics underlying cardiomyocyte responses to hypertrophic stimuli and also other cardiomyocytes, including adult and iPS-derived cardiomyocytes [21]. Our results provide evidence that this imaging system can reliably detect dynamic increases in cardiomyocyte surface area and volume that accompany the hypertrophic response to the model stimulus, norepinephrine (Figure 2C-F). This has significant potential to enable future studies to explore the temporal aspects of cardiomyocyte hypertrophic responses. Additionally, these studies could further exploit the advantages of this approach to characterize cardiomyocyte responses to various hypertrophic inducers and investigate potential synergies between them. However, our results also shed light on the technological limitations of the approach that should be considered.

Single-cell tracking using the HOLOMONITOR App Suite software revealed a statistically significant decrease in the starting optical volumes of the norepinephrine-stimulated cardiomyocytes (Figures 1D and 2D). It is unlikely that the cardiomyocyte volumes truly differed between the untreated and treated cells at the start of the experiment since the cardiac cells in both conditions were derived from the same tissue preparations. While we attempted to start our digital holographic imaging time-course experiments soon after norepinephrine treatment, the time it took to initiate our time-lapse movies after treatment ranged from 1 to 2 h due to technical obstacles. These include the time it takes to reacclimatize cultures back to 37 °C to resolve condensation on the culture plate imaging surface, which interferes with image acquisition. Additional time is also spent operating the analysis software and identifying adequate imaging fields prior to starting time-lapse acquisition. Our results show that norepinephrine has a strong cardiomyocyte cell spreading effect by 24 h (Figure 1C). Therefore, it is possible that cardiomyocytes may have already started to spread soon after norepinephrine stimulation during our 1–2 h transition time prior to image acquisition. This would likely impact the fidelity of image segmentation and cardiomyocyte identification since it is more difficult for the analysis software to distinguish signal from background noise in thinner cellular processes than in thicker regions of the cell (Figure S1). Compared to cells exhibiting a more compact and rounded morphology, this error would be magnified in cardiomyocytes that strongly spread over the substratum, which would lead to an underestimation of volume measurements. Therefore, cell spreading could be a confounding factor when interpreting raw unnormalized morphology measurements observed between control and treatment conditions and should be accounted for in the experimental design. This cautions against the interpretation of raw morphological measurements as absolute since the accuracy of these measurements is dependent on segmentation accuracy and cell thickness (Figure 2C,D). Nevertheless, the approach may be useful for gaining insights into the relative dynamics of underlying hypertrophic processes (Figure 2E,F).

Another limitation of the technology is that accurate image segmentation and the identification of individual cardiomyocytes requires well-isolated cells that are not integrated into clusters. To accommodate this technological caveat, we seeded cardiac cultures at moderate densities and focused our analyses on individual cardiomyocytes away from tight cell clusters in order to support the fidelity of our single-cell quantifications. In vivo, however, cardiomyocytes are tightly associated with each other through gap junctions that facilitate the propagation of electrical signals to regulate contractility [22]. Therefore, it is important to note that cardiomyocyte hypertrophic responses may differ in isolated cells compared to those tightly integrated within tissues.

Nevertheless, the Holomonitor M4 is an accessible, compact, digital holographic imaging microscope that supports the quantitative three-dimensional label-free imaging of cultured cells. In this study, we demonstrate the potential of this technology to provide novel insights into the timing and dynamics of cardiomyocyte hypertrophic growth. However, the advantages of digital holographic imaging have the potential to support the discovery of new aspects in cardiomyocyte cell biology as well.

## 5. Conclusions

Our results demonstrate the feasibility of using the Holomonitor M4 digital holographic imaging microscope to monitor dynamic changes in cardiomyocyte surface area and volume in response to norepinephrine-induced hypertrophic growth. They highlight the potential of this label-free imaging approach to characterize cellular dynamics during cardiomyocyte hypertrophic responses. We observed dynamic increases in cardiomyocyte surface area and volume following norepinephrine stimulation, validating the imaging system’s ability to detect hypertrophic changes over time. However, we also identified technological limitations. These challenges include the limitations of accurate image segmentation within tightly clustered cardiomyocytes and the impact of cell spreading on the ability of the software to detect signals from background noise accurately. Despite these limitations, the Holomonitor M4 offers a promising tool for exploring the temporal aspects of cardiomyocyte hypertrophy, which may provide novel insights into the progression of pathological hypertrophic events. Further refinements in experimental protocol will likely help maximize the benefits of this digital holographic imaging approach to characterize cardiomyocyte hypertrophy dynamics in vitro.

## Supplementary Material

Movie S1

Movies S2

Figure S1

## Data Availability

The original contributions presented in the study are included in the article/supplementary material, further inquiries can be directed to the corresponding author.
